# Peripheral nervous system: A promising source of neuronal progenitors for central nervous system repair

**DOI:** 10.3389/fnins.2022.970350

**Published:** 2022-07-29

**Authors:** Jessica L. Mueller, Rhian Stavely, Ryo Hotta, Allan M. Goldstein

**Affiliations:** Department of Pediatric Surgery, Massachusetts General Hospital, Harvard Medical School, Boston, MA, United States

**Keywords:** regenerative therapies, central nervous system, stem cells, autologous cells, neurologic disorders, peripheral nervous system

## Abstract

With a steadily aging population there is an increasing prevalence of neurological disorders. Given the lack of effective treatment strategies and a limited ability for the central nervous system (CNS) to regenerate endogenously, there is a critical need to better understand exogenous strategies for nervous system repair. Stem cell therapy offers a promising approach to promote the repair of neurologic tissue and function, however studies to date have been limited by various factors including challenges in harvesting donor cells from the CNS, ethical concerns regarding use of embryonic or fetal tissue, tumorigenic potential of induced pluripotent stem cells, and immune-mediated rejection of non-autologous cell sources. Here we review and propose two alternative sources of autologous cells derived from the peripheral nervous system (PNS) for CNS repair: enteric neuronal stem cells (ENSCs) and neural crest-derived Schwann cells found in subcutaneous adipose tissue (termed SAT-NSCs). ENSCs can be successfully isolated from the postnatal enteric nervous system, propagated *in vitro*, and transplanted successfully into models of CNS injury via both direct intracerebral injection and systemic tail vein injection. Similarly, SAT-NSCs can be readily isolated from both human and mouse adipose tissue and, although not yet utilized in models of CNS injury, have successfully been transplanted and restored function in models of colonic aganglionosis and gastroparesis. These unique sources of PNS-derived autologous cells offer an exciting option for stem cell therapies for the CNS as they have proven neurogenic potential and eliminate concerns around tumorigenic risk, ethical considerations, and immune-mediated rejection.

## Introduction

When damaged, the central nervous system (CNS) has a limited ability to regenerate endogenously. This is clinically important as it results in ineffective treatment strategies for most diseases involving CNS injury. With a steadily aging population, there is an increasing prevalence of CNS disorders, including neurodegenerative diseases, traumatic brain injury, neurotoxicity from cancer therapy, and spinal cord injury. Given the significant public health burden of neurologic diseases and the dearth of effective therapies, recent efforts have focused on regenerative therapy via exogenous stem-cell based therapies (Alessandrini et al., 2018; Andrzejewska et al., 2021).

Stem cells have the unique ability to self-renew and to differentiate into multiple different lineages (Venkei and Yamashita, 2018). Stem cell therapy, or treatment of affected tissue with these regenerative progenitor cells, offers an exciting approach to promote repair of neurologic tissue and restore function in CNS diseases. However, thus far, studies have been limited by various factors including challenges gathering donor cells directly from the CNS, ethical concerns with use of embryonic or fetal tissue, tumorigenic potential of induced pluripotent stem cells, and immune-mediated rejection of non-autologous cell sources.

**FIGURE 1:**
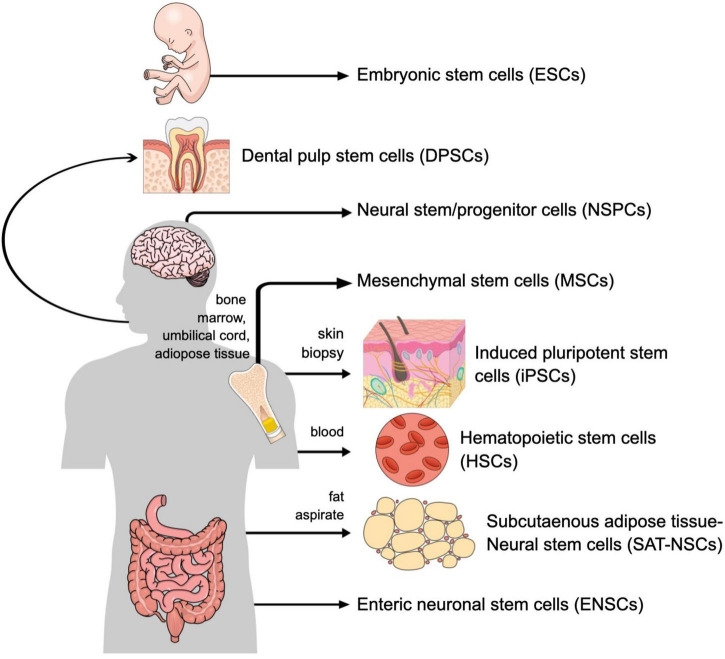
Cell sources for regenerative central nervous system (CNS) therapy.

There are numerous candidate donor cells that have been studied in the context of CNS injury (Table 1 and Figure 1). Neural stem/progenitor cells (NSPCs), which are harvested directly from the neural tissue itself, when utilized in models of spinal cord injury can replace lost neurons and glia and promote a local environment of growth and regeneration (Mothe and Tator, 2013). NSPCs can be harvested from both adult and embryonic brain and spinal cord tissue. These cells are widely considered the ideal cell type for use in CNS injury because they most closely resemble the cells being replaced and may therefore respond best to signals from the surrounding local environment. Unfortunately, NSPCs are located deep within the brain so their harvest requires an invasive procedure (Deng et al., 2018). To overcome the challenge of harvesting donor cells directly from the CNS, efforts have focused on embryonic stem cells (ESCs) and induced pluripotent stem cells (iPSCs). Use of embryonic stem cells in spinal cord injury showed significant potential as they were able to engraft, promote axonal growth, undergo remyelination, promote angiogenesis, and recover locomotor function (Kumagai et al., 2009). Similarly, in both rat (Andres et al., 2011) and gerbil (Ishibashi et al., 2004) models of stroke injury, transplantation of fetal-derived neural progenitor cells resulted in improved axonal rewiring and axonal transport as well as enhanced functional recovery. However, the use of embryonic or fetal tissue is controversial and raises ethical concerns. Additionally, treatment with non-autologous cell sources requires immunosuppressive medications, which are associated with numerous risks, including higher rates of infectious diseases, increased development of lymphoproliferative disorders and cancers, and medication specific toxicities. Furthermore, immune cell infiltration following neurotrauma and during neurodegeneration promotes CNS protection and repair (Schwartz and Moalem, 2001; Simard et al., 2006; Ziv et al., 2006; Beers et al., 2008), and this may be inhibited by the use of immunosuppression (Kulbatski, 2010).

**TABLE 1:** Cell sources for regenerative central nervous system (CNS) therapy.

Source of cells	Accessibility	Ethical concerns	Tumorigenic	Autologous	Nervous system-derived
NSPCs	Low	Moderate	Sometimes	Sometimes	Yes
ESCs	Low	Significant	Yes	No	No
iPSCs	High	None	Yes	Yes	No
MSCs	High	None	No	Yes	No
HSCs	High	None	No	Yes	No
DPSCs	High	None	No	Yes	No
ENSCs	High	None	No	Yes	Yes
SAT-NSCs	High	None	No	Yes	Yes

NSPCs, neural stem/progenitor cells; ESCs, embryonic stem cells; iPSCs, induced pluripotent stem cells; MSCs, mesenchymal stem cells; HSCs, hematopoietic stem cells; DPSCs, dental pulp stem cells; ENSCs, enteric neuronal stem cells; SAT-NSCs, subcutaneous adipose tissue–neural stem cells.

Induced pluripotent stem cells, in which somatic cells are reprogrammed via the introduction of specific transcription factor genes to become pluripotent stem cells that exhibit the proliferation and differentiation capacity analogous to embryonic stem cells, bypass these ethical concerns. These cells are also easily obtained from skin biopsies and are autologous, avoiding some of the accessibility and immunogenic concerns of the cell sources described above. Application of iPSCs in models of spinal cord injury and stroke have shown successful engraftment and improved functional recovery (Oki et al., 2012; Nakamura and Okano, 2013), but there have been concerns regarding their tumorigenic potential (Nakamura and Okano, 2013; Deng et al., 2018).

Given the current obstacles hindering stem cell therapy in the CNS, identification of a readily available, not genetically reprogrammed, and autologous source of neural progenitor cells would be optimal. Other autologous sources of stem cells have been proposed and reviewed, including mesenchymal stem cells (MSCs), hematopoietic stem cells (HSCs), and dental pulp derived stem cells (DPSCs). MSCs can be obtained from multiple sources, including bone marrow, umbilical cord blood, and adipose tissue and demonstrate a high differentiation plasticity including the ability to differentiate into mesodermal lineages, including osteocytes, adipocytes, and chondrocytes, as well as reported abilities to differentiate into non-mesodermal lineages, including the neuronal linage (Hernández et al., 2020). They have been reviewed previously and have shown promise in multiple clinical trials of neurodegenerative diseases, including amyotrophic lateral sclerosis, Alzheimer’s disease, and Parkinson’s disease (Ullah et al., 2015; Andrzejewska et al., 2021). HSCs can be obtained via peripheral blood, and have been primarily studied in the context of autoimmune neurologic disorders, particularly multiple sclerosis (Balassa et al., 2018; Massey et al., 2018). Dental pulp derived stem cells have been isolated from humans (Gronthos et al., 2000; Nuti et al., 2016) and successfully transplanted into a rat model of spinal cord injury, demonstrating regeneration of transected axons and recovery of hindlimb motor function (Sakai et al., 2012), and have shown promise in models of both retinal and CNS injury and disease (Mead et al., 2017).

Although these cell sources have shown great potential in models of CNS disease, they are not derived from a neuronal niche. Moreover, in the case of MSCs, their therapeutic effects are predominantly reliant on paracrine signaling and neurotrophic support of the injured nervous system, with limited cell survivability. In this review, we therefore propose two alternative sources of autologous cells for CNS repair, both directly derived from the peripheral nervous system and therefore with intrinsic neurogenic potential: enteric neuronal stem cells (ENSCs) and neural crest-derived Schwann cells found in subcutaneous adipose tissue (termed SAT-NSCs).

## Enteric neuronal stem cells

The enteric nervous system (ENS) is an extensive network of neurons and glia within the wall of the gastrointestinal (GI) tract that regulates many of the GI functions independently from CNS input (Furness, 2012). It has been shown that neural progenitors can be isolated from the GI tract of embryonic and postnatal rodents (Kruger et al., 2002; Almond et al., 2007). Subsequently, these ENSCs have been identified in human gut tissue ranging from embryonic stages to late adulthood, including even an octogenarian (Metzger et al., 2009a). They are easily accessible and have been successfully isolated from neonates (Almond et al., 2007; Lindley et al., 2008), children (Rauch et al., 2006), and adults (Metzger et al., 2009a), from both the small and large intestine (Cheng et al., 2016), and from full-thickness biopsies and mucosa (Metzger et al., 2009b). Importantly, these cells can be harvested using minimally invasive techniques, including endoscopy (Metzger et al., 2009b). Enteric neuronal stem cells can be propagated *in vitro* as floating neurospheres (clusters of concentrated NSCs) and expanded exponentially (Rauch et al., 2006; Lindley et al., 2009; Metzger et al., 2009a; Cheng et al., 2017). This expansion step is crucial, as it allows a significant increase in the size of the donor cell pool without having to harvest more cells. These neurospheres contain ENSCs that give rise to neurons and glial cells following their transplantation to the embryonic (Lindley et al., 2009; Cheng et al., 2017) and postnatal (Hotta et al., 2013; Hetz et al., 2014; Cheng et al., 2017) mouse gut and restore GI motility in mice with enteric neuropathies (Lindley et al., 2008; McCann et al., 2017). ENSCs have also been transplanted into aganglionic mouse colon generated using diphtheria-toxin mediated ENS ablation (Bhave et al., 2019), where successful cell engraftment, migration, and differentiation of transplanted ENSCs were observed. More importantly, transplantation of ENSCs restored gut architecture changes and reduced mucosal inflammation (Bhave et al., 2019). To build upon the success of ENSCs as a stem cell source for treating enteric neuropathies, two studies have demonstrated their application for the treatment of brain injury (Osman et al., 2014; Belkind-Gerson et al., 2016). ENSCs were transplanted directly into the brains of mice following blunt injury (Belkind-Gerson et al., 2016) or radiation-induced injury (Osman et al., 2014; Belkind-Gerson et al., 2016). The cells survived at least 4 weeks following transplantation, differentiated into neurons and glia, and modulated the local environment to stimulate endogenous neurogenesis (Belkind-Gerson et al., 2016). Additionally, ENSCs delivered via tail vein injection were found to home to the site of injury in the brain where they survived for 10 weeks post-injection, and similarly stimulated endogenous neurogenesis (Belkind-Gerson et al., 2016). The success of systemic injection is clinically appealing compared to a direct approach to the brain or spinal cord.

Other studies have found similar success using ENSCs in models of spinal cord injury (Jevans et al., 2018, 2021). ENSCs were first co-cultured *in vitro* with spinal cord-derived cells, which revealed the formation of extensive cellular connections between the ENSCs and spinal cord-derived cells, as well as the presence of differentiated TuJ1+ neurons, S100+ glia, and Sox10+ stem cells within the transplanted neurospheres. Furthermore, following *in vivo* transplantation of ENSCs to an ablated region of chick spinal cord, donor ENSCs were found to form bridging connections within the injury zone up to 12 days later (Jevans et al., 2018). Combined *in vivo* treatment with ENSCs and chondroitinase ABC, an enzyme that breaks down chondroitin sulfate proteoglycans, which play a role in scar formation following injury, revealed improved regenerative effects compared to treatment with stem cells alone (Jevans et al., 2021). These studies suggest that transplantation of ENSCs can be an exciting treatment option for repair in CNS disorders, particularly when combined with other therapies to enhance their regenerative abilities.

## Neural crest-derived Schwann cells in subcutaneous adipose tissue

Adipose tissue has been studied extensively as a potential source for cell based therapies since the isolation of progenitors with mesenchymal trilineage and neuronal differentiation potential were first reported 20 years ago (Zuk et al., 2001, 2002). Specifically, the SAT contains a reservoir of adipose stem cells within the stroma that can be easily obtained via minimally invasive techniques, including simple aspiration or suctioning of the fatty tissue (Zuk et al., 2002). This heterogenous population of progenitor cells are often referred to collectively as mesenchymal stem cells or multipotent stromal cells (MSCs) due to their differentiation potential and presumptive stromal cell origin. From 2007 to 2019 there were over 270 clinical trials worldwide studying this cell population in a variety of diseases, which overall suggested a favorable patient safety profile (Chu et al., 2019). Adipose stem cells have been primarily studied in the context of regenerative medicine and numerous inflammatory diseases including osteoarthritis, degenerative arthritis, cartilage or tendon injury, graft-vs.-host diseases, and chronic kidney diseases (Peng et al., 2019). They have not been well studied in the context of neurologic diseases (Hernández et al., 2020). Recent trials examining the safety of intracerebroventricular (ICV) injection of autologous adipose-derived stromal vascular fraction cells indicate favorable safety profiles across various disease states (24 subjects across 7 diseases) and promising clinical outcomes in the limited data for subjects with Alzheimer’s disease (*n* = 10) and progressive multiple sclerosis (*n* = 6) (Duma et al., 2019). It was estimated that only 7.5% of the injected cells are adipose stem cells; therefore, these results could be improved upon by administering a more homogenous stem cell population.

Importantly, adipose stem cells can be cultured in conditions favoring the production of a cell population consistent with neural progenitor cells (Zuk et al., 2002; Peterson et al., 2018; Peng et al., 2019) that display a phenotype similar to stem cells derived directly from embryonic brain (Peterson et al., 2018). These adipose-derived neural progenitor cells are thought to arise from the transdifferentiation of isolated MSCs which acquire neural progenitor traits including propagation in culture as neurospheres, and can be induced in neuronal media conditions to become neurons and glia (Peng et al., 2019). When differentiated into neurons, cells derived from adipose tissue have network characteristics and spontaneous spiking activity similar to primary neuronal cultures (Peterson et al., 2018), suggesting their ability to form a functioning nervous system.

Despite this knowledge of a neural progenitor population within adipose tissue, there have been few studies to date on adipose stem cells in the context of neurologic diseases. In a mouse stroke model, adipose-derived stem cells were found to reduce the size of the infarct and increase neurologic recovery via decreasing autophagy (Kuang et al., 2020). Another study found that the early delivery of adipose-derived stem cells combined with a rehabilitation program improved behavioral recovery, but not infarct size, in a rat stroke model (Mu et al., 2019). Our lab recently studied adipose-derived stem cells in diseases involving the enteric nervous system (Stavely et al., 2022). Importantly, while previously it was thought that the neural progenitors found within adipose tissue were derived from MSCs, we determined that these neural progenitors are likely to be NSCs that are distinct from adipose-derived MSCs and reside within the local nervous system niche of the SAT. These cells become transcriptionally distinct from Schwann cells and acquire features of NSCs during *in vitro* culture, which we anticipate is essential for their expansion and differentiation potential. Following transplantation into the gastrointestinal tract, these SAT-NSCs can successfully engraft, migrate within the muscularis layer, and differentiate into enteric neurons and glia. Transplantation of these cells directly into the gastric antrum in a mouse model of gastroparesis improved gastric emptying of both liquids and solids. Furthermore, transplantation of these cells into the aganglionic distal colorectum of a mouse model of Hirschsprung disease showed successful engraftment, migration, and survival of SAT-NSCs two to 3 weeks after surgery, as well as restoration of neural-evoked smooth muscle contractility. This evidence of functional recovery suggests that SAT-NSCs represent a source of autologous NSCs that could be used for treating enteric nervous system disorders. Given the exciting therapeutic potential of SAT-NSCs in ENS disorders, we believe that these cells offer significant potential for treatment of CNS disorders and further research is warranted.

## Discussion

The above data reveal the exciting potential of two alternative sources of autologous stem cells, both of which are easily accessible and can be harvested from the donor with minimal risk. Importantly, they are derived from the nervous system and therefore possess intrinsic neurogenic potential that can be leveraged for CNS applications. ENSCs have been shown numerous times to be easily accessible from donors of all ages, from both small and large intestine, and from full-thickness and mucosal biopsies, making them a feasible source of donor cells for regenerative cell therapy. They engraft, migrate, differentiate, and survive when transplanted into the gut, and have shown restoration of normal gut architecture and improved survival when transplanted into models of ENS injury. Moreover, these cells have been shown to engraft, migrate, differentiate, survive, and promote endogenous neurogenesis when injected intracerebrally into areas of injured brain or when delivered systemically. Furthermore, ENSCs have also successfully engrafted, differentiated, and survived in *in vitro* and *in vivo* models of spinal cord injury. Further research is needed to assess functional recovery following ENSC transplantation in models of CNS injury.

Neural crest-derived Schwann cells found in subcutaneous adipose tissue are even more easily accessible than ENSCs, and can be isolated via simple aspiration or liposuction. These cells can similarly be expanded in culture to form neurospheres, and can engraft, migrate, undergo neuroglial differentiation, and survive when transplanted directly into the gut. Importantly, these cells form functional neuronal networks, as evidenced by the functional recovery following transplantation in two models of ENS disease, gastroparesis and Hirschsprung disease. Given this success, we believe SAT-NSCs offer great potential for treatment of CNS diseases, and further research is certainly warranted. These unique sources of PNS-derived autologous cells offer an exciting option for regenerative cell therapy in the CNS.

## Author contributions

JM wrote the manuscript and created the table and figure. RH, RS, and AG helped write the manuscript and reviewed the table and figure. All authors contributed to the article and approved the submitted version.
